# ﻿Two new species of the genus *Halichoanolaimus* (Nematoda, Selachinematidae) from the intertidal zone of the Yellow Sea, China

**DOI:** 10.3897/zookeys.1208.124047

**Published:** 2024-07-31

**Authors:** Mian Huang, Hongxiu Zhai

**Affiliations:** 1 College of Life Sciences, Liaocheng University, Liaocheng 252059, China Liaocheng University Liaocheng China

**Keywords:** Biodiversity, free-living marine nematode, *Halichoanolaimussinensis* sp. nov., *Halichoanolaimuszhangi* sp. nov., identification key, taxonomy

## Abstract

Two new marine nematode species belonging to the genus *Halichoanolaimus* from the intertidal zone of the Yellow Sea are described. *Halichoanolaimussinensis***sp. nov.** is characterized by amphideal fovea with 2.5–3.0 turns, 20–27% of corresponding body diameters; spicules curved, middle portion broad, tapering distally, 1.4–1.5 cloacal body diameters long; gubernaculum slender consisting of two detached lateral pieces tapering distally; 10–13 papilliform precloacal supplements in two groups, the posterior three supplements smaller and closer to each other, the remaining supplements larger and widely spaced; tail conico-cylindrical with a half cylindrical portion. The second new species, *Halichoanolaimuszhangi***sp. nov.** is distinct by having lateral differentiation present except in anterior half of pharynx which has even punctations, amphideal fovea with 3.0–3.3 turns, spicules curved, gradually narrowing from proximal to distal end with pointed tip, 7 papilliform precloacal supplements, gradually increasing the spacing distance forward, two rows of subventral conical setae situated at the precloacal region, tail elongated, filiform. An updated key to 30 valid species of *Halichoanolaimus* is provided.

## ﻿Introduction

The Yellow Sea is located on the edge of the western Pacific Ocean, between the Chinese mainland and the Korean Peninsula. It is a semi-enclosed inland shallow sea basin. Biodiversity surveys and taxonomical studies on nematodes in the Yellow Sea have been carried out in recent years. More than 350 species of nematodes have been identified, of which 105 species were new to science (Hao et al. 2022; Chu et al. 2023). The new species accounted for 30% of the known species. However, the total number of nematodes in this sea area is unknown, and new species are routinely found. It is, therefore, important to continue investigating the taxonomy of nematodes in the region.

The genus *Halichoanolaimus* was established by de Man (1886) with the type species of *H.robustus* (Bastian, 1865). It is a common and diverse genus of predatory nematodes belonging to the family Selachinematidae and found from shallow seas to the abyssal plain (Miljutin et al. 2010; Leduc 2020). The most recent species descriptions were provided by Xiao and Guo (2023). Based on the reviews by Tchesunov (2014), Leduc and Zhao (2016), Leduc (2020) and Huang and Guo (2022), *Halichoanolaimus* is characterized by a cuticle with lateral differentiation in the form of larger and more widely spaced punctations; all anterior sensilla papilliform; a buccal cavity consisting of two parts separated by a row of teeth; pharynx without bulb; precloacal supplements usually papilliform or setiform and tail conico-cylindrical, elongated with a distal filiform portion, or conical. Within *Halichoanolaimus*, the species are distinguished by rather few main characters: the number of amphidial turns, number and arrangement of precloacal supplements, structure of spicules and gubernaculum, and shape and length of the tail. A key to the identification of 22 valid species for the genus was given by Zograf et al. (2015). Subsequently, six species, *H.anisospermus* Leduc & Zhao, 2016, *H.stagnalis* Gagarin & Long, 2017, *H.funestus* Leduc, 2020, *H.ossilagulus* Leduc, 2020, *H.pumilus* Leduc, 2020 and *H.sicaoensis* Xiao & Guo, 2023, were described. To date, 28 valid species within *Halichoanolaimus* have been recorded worldwide.

## ﻿Materials and methods

In order to investigate the diversity of free-living nematodes along the coast of the Yellow Sea, China, sediment samples were collected in several intertidal sites in 2008 and 2022 respectively. The meiofauna samples were obtained from the top sediment layer (0–8 cm deep) using a 2.9 cm diameter sawn-off syringe. The samples were fixed with an equal amount of 10% formalin solution.

In the laboratory, samples were stained with 0.1% Rose Bengal for more than 12 hours (Higgins and Thiel 1988). The stained samples were poured into two layers of sieves (500 and 30 µm mesh sizes) respectively, and washed with tap water to remove silt and separate macrofauna from meiofauna. The heavier sediment particles retained on the 30-micrometer mesh were removed using centrifugation with Ludox-TM colloidal silica (50% colloidal silica suspension in water; Sigma Aldrich Co., USA) with a specific gravity of 1.15 g/ml (de Jonge and Bouwman 1977). Each sample was washed into a Petri dish with distilled water and meiofauna was sorted under a stereoscopic microscope (Olympus SZ 51). Nematodes were transferred into a 9:1 (v/v) solution of 50% alcohol-glycerol in an embryo dish to slowly evaporate to pure glycerol, and then mounted into permanent slides (McIntyre and Warwick 1984).

Finally, the specimens were mounted in glycerin on permanent slides. Observation and measurement were carried out using a differential interference contrast microscope (Leica DM 2500) and Leica software of LAS X version 3.3.3. Line drawings were made with the aid of a camera lucida. Type specimens were deposited in the Marine Biological Museum of the Chinese Academy of Sciences, Qingdao.

Abbreviations are as follows: **a**, the ratio of body length to maximum body diameter; **abd**, body diameter at cloaca or anus; **b**, ratio of body length to pharynx length; **c**, ratio of body length to tail length; **cbd**, corresponding body diameter; **c**′, ratio of tail length to cloacal or anus body diameter; **V**%, position of vulva from anterior end expressed as a percentage of total body length.

## ﻿Results and discussion

### ﻿Taxonomy

**Class Chromadorea Inglis, 1983**

**Order Chromadorida Chitwood, 1933**

**Family Selachinematidae Cobb, 1915**

#### ﻿Genus Halichoanolaimus

Taxon classificationAnimaliaChromadoridaSelachinematidae

De Man, 1886

E03A1EC4-C0E8-5EEA-AC2F-F970BD25D6A1

##### Diagnosis

**(modified from Leduc 2020).** Cuticle with lateral differentiation in the form of larger and more widely spaced punctations. All anterior sensilla usually papilliform. Buccal cavity separated into two chambers by transversal sets of denticles; posterior chamber of buccal cavity surrounded by three Y-shaped pairs of cuticularized rhabdions. Pharynx without anterior or posterior bulb. Intestine of adult stages blind. Precloacal supplements usually papilliform or setiform. Tail with conical proximal portion and often elongated cylindrical distal portion.

### ﻿Valid species list

*Halichoanolaimusanisospermus* Leduc & Zhao, 2016

*Halichoanolaimusbalochiensis* Turpeenniemi, Nasira & Maqbool, 2001

*Halichoanolaimusbispirae* Daschenko & Belogurov, 1991

*Halichoanolaimusbrandtae* Zograf, Trebukhova & Pavlyuk, 2015

*Halichoanolaimuscaucasicus* Sergeeva, 1973

*Halichoanolaimuschordiurus* Gerlach, 1955

*Halichoanolaimusconsimilis* Allgén, 1933

*Halichoanolaimusdolichurus* Ssaweljev, 1912

*Halichoanolaimusduodecimpapillatus* Timm, 1954

*Halichoanolaimusfunestus* Leduc, 2020

*Halichoanolaimuslanceolatus* Vitiello, 1970

*Halichoanolaimuslukjanovae* Sergeeva, 1973

*Halichoanolaimusmacrophallus* Gourbault & Vincx, 1985

*Halichoanolaimusmacrospiculatus* Hopper, 1961

*Halichoanolaimusminor* Ssaweljev, 1912

*Halichoanolaimusminutissimus* Timm, 1961

*Halichoanolaimusnorvegicus* Allgén, 1940

*Halichoanolaimusossilagulus* Leduc, 2020

*Halichoanolaimusovalis* Ditlevsen, 1921

*Halichoanolaimuspossjetiensis* Belogurov & Fadeeva, 1980

*Halichoanolaimuspumilus* Leduc, 2020

*Halichoanolaimusquattuordecimpapillatus* Chitwood, 1951

*Halichoanolaimusraritanensis* Hasbrouck, 1966

*Halichoanolaimusrobustus* (Bastian, 1865) de Man, 1886

*Halichoanolaimussicaoensis* Xiao & Guo, 2023

*Halichoanolaimussonorus* Belogurov & Fadeeva, 1980

*Halichoanolaimusstagnalis* Gagarin & Long, 2017

*Halichoanolaimusunicus* Inglis, 1968

#### ﻿ Halichoanolaimus sinensis sp. nov.

Taxon classificationAnimaliaChromadoridaSelachinematidae

787E262F-B526-56F2-B2AB-BACADACF5638

https://zoobank.org/3BCBB4D2-AED6-4A98-81B5-F637EA95290F

**Figure 1.**
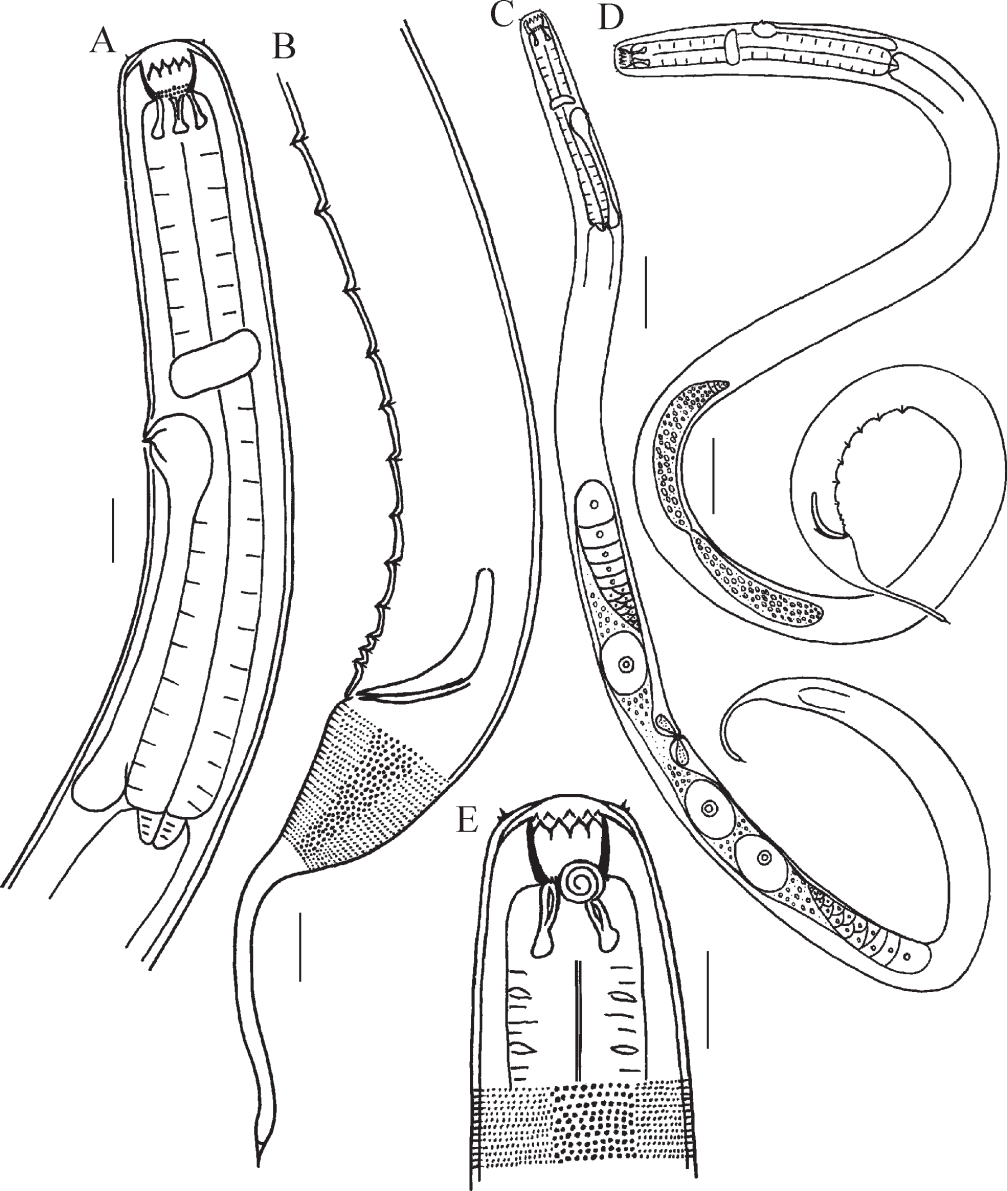
Drawings of *Halichoanolaimussinensis* sp. nov. **A** pharyngeal region of male **B** posterior end of male **C** entire body of female **D** entire body of male **E** anterior end of male. Scale bars: 30 μm (**A, B, E**); 100 μm (**C, D**).

**Figure 2.**
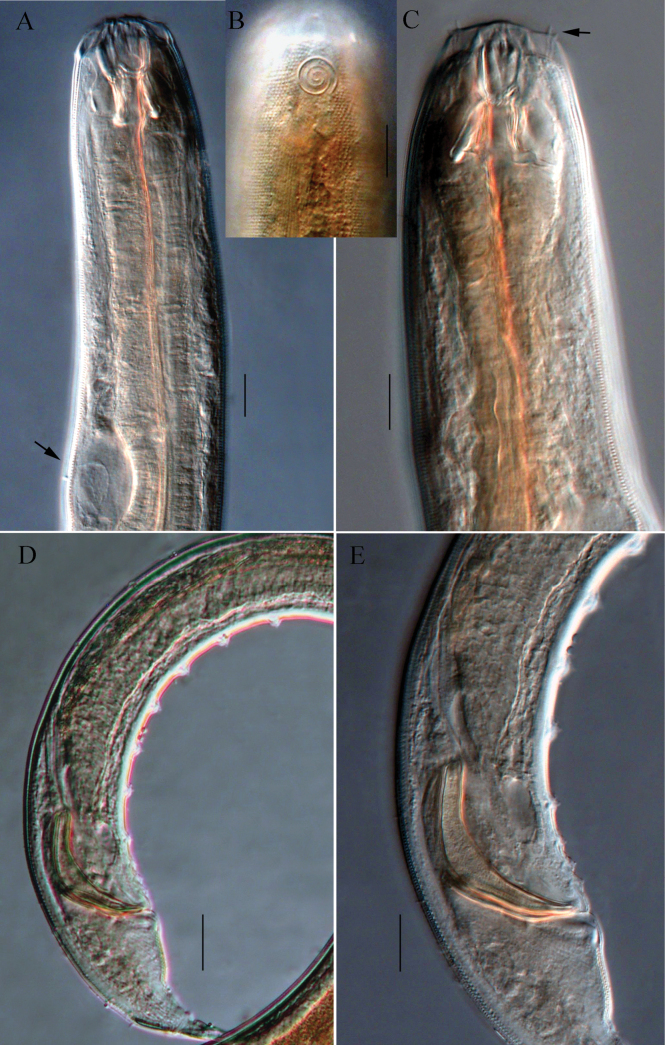
Microscopic images of *Halichoanolaimussinensis* sp. nov. **A** anterior end of holotype, showing buccal cavity and excretory ampulla (arrow) **B** anterior end of male 2, showing amphidial fovea **C** anterior end of holotype, showing stomatorhabdions and anterior sensilla (arrow) **D** posterior part of holotype, showing supplements **E** cloacal region, showing spicules, gubernaculum and supplements. Scale bars: 20 μm (**A, B, C, E**); 30 μm (**D**).

Figs 1 , 2 , Table 1

##### Material examined.

Four males and two females were obtained. ***Holotype***: ♂1 on slide RZ08-7-5; ***paratypes***: ♂2 on slide RZ08-7-2, ♂3 on slide RZ08-7-5, ♂4 and ♀1 on slide RZ08-7-3, and ♀2 on slide RZ08-7-2. Type specimens were deposited in the Marine Biological Museum of the Chinese Academy of Sciences, Qingdao.

##### Type locality and habitat.

Holotype and all additional specimens were found from intertidal silt sediment at Rizhao coast of the Yellow Sea; 35°26'N, 119°34'E; 0–2 cm and 2–5 cm sediment depth.

##### Etymology.

The specific epithet refers to the country origin, China.

##### Measurements.

All measurement data are given in Table 1.

**Table 1.** Individual measurements of *Halichoanolaimussinensis* sp. nov. (in µm except for ratios; -, null).

Characters	Holotype	Paratypes	
	male	males (*N* = 3)	females (*N*= 2)
Total body length	2416	2197±59.0 (2138–2259)	2792±38.2 (2765–2819)
Maximum body diameter	66	72.3±2.1(70–74)	86.0±5.7 (82–90)
Head diameter	36	35.3±3.1 (32–38)	39.0±0.8 (37–41)
Length of outer labial sensilla	4	3.0±0 (3–3)	3.0±0.0 (3–3)
Depth of buccal cavity	46	36.0±2.6 (31–38)	40.0±0.0 (40–40)
Width of amphid	14	11.3±1.2 (10–12)	10.0±0.0 (10–10)
Amphid from anterior end	20	20.0±1.7 (19–22)	14.0±1.4 (13–15)
Nerve ring from anterior end	140	117±0 (117–117)	–
Length of pharynx	320	301.3±24.0 (274–319)	307.0±18.4 (294–320)
Body diameter at pharyngeal base	66	67.0±4.0 (63–71)	69.0±9.9 (62–76)
Spicule length along arc	82	79.7±5.5 (76–86)	–
Length of gubernaculum	42	45.0±5.3 (41–51)	–
Number of precloacal supplements	10	12.0±1.0 (11–13)	–
Vulva from anterior end	–	–	1251.5±153.4 (1143–1360)
V%	–	–	44.8±4.9 (41.3–48.2)
Body diameter at cloaca	55	55.3±1.2 (54–56)	–
Tail length	166	190.7±7.6 (182–196)	204.0±2.8 (202–206)
a	36.6	30.4±1.3 (28.9–31.4)	32.6±2.6 (30.7–34.4)
b	7.6	7.3±0.8 (6.9–8.2)	9.1±0.4 (8.8–9.4)
c	14.6	11.5±0.8 (10.9–12.4)	13.7±0.0 (13.7–13.7)
c′	3.0	3.5±0.2 (3.3–3.6)	3.1±0.4 (2.8–3.4)

##### Description.

**Males.** Body cylindrical, tapering slightly towards posterior end. Cuticle with transverse rows of punctations, lateral differentiation consisting of slightly larger and more widely spaced punctations. Cuticle pore not observed. Somatic setae short, 3 µm long, sparsely distributed. Lip region slightly rounded. Six inner labial sensilla papillose; six outer labial sensilla setiform, 3–4 µm long, at same level as four papilliform cephalic sensilla (Fig. 2C). Amphideal fovea multispiral with 3 turns (Fig. 2B), 25–27% of corresponding body diameter in width, located at the level of the middle of buccal cavity, ca 20 µm from anterior end of body. Buccal cavity large, ca 45 µm deep, divided into anterior and posterior portions by two rows of 15–17 denticles. Anterior portion of buccal cavity cup-shaped, with three sets of three cuticularized rhabdions; posterior portion of buccal cavity narrower, cylindrical, surrounded by three Y-shaped pairs of cuticularized rhabdions with swollen bases, 20 µm long. Pharynx cylindrical, anterior end swelling, wrapped the buccal cavity, without posterior bulb. Pharyngeal lumen cuticularized. Nerve ring at ca 44% of pharynx length from anterior end. Secretory-excretory system present. Renette cell small, situated at level of cardia; ampulla large, excretory pore situated slightly posterior to the nerve ring, ca 180 µm from the anterior end. Cardia small, surrounded by intestine.

Reproductive system diorchic with two opposed, outstretched testes. Anterior testis to the right or ventrally to intestine, posterior testis to the left side of intestine. Spicules paired, curved, middle portion broad, tapering distally, 1.4–1.5 cloacal body diameters long, interior of spicules granular in appearance. Gubernaculum slender, consisting of two detached lateral pieces tapering distally, adjoining the dorsal side of spicules. 10–13 papilliform precloacal supplements in two groups, the posterior three supplements smaller and closer to each other (Fig. 2E), located 7 µm from the cloaca; 5 µm from each other; remaining supplements larger and widely spaced, ca 25 µm from each other. Each supplement consisting of conical papilla and an internal duct. Tail conico-cylindrical with posterior cylindrical portion comprising about half of total tail length. Caudal setae absent. Three caudal glands located posterior to spicules, spinneret present, 7 µm long.

**Females.** Similar to males, but with slightly larger body and slightly smaller amphideal fovea (20% of corresponding body diameter in width and with 2.5 turns). Reproductive system didelphic, with two opposed, reflexed ovaries. Anterior ovary to the left of intestine and posterior ovary to the right of intestine. Vulva situated slightly pre-median. Intestine blind, anus not observed.

##### Differential diagnosis and discussion.

*Halichoanolaimussinensis* sp. nov. is characterized by amphideal fovea with 2.5–3.0 turns, 20–27% of corresponding body diameter; spicules curved, middle portion broad, tapering distally, 1.4–1.5 cloacal body diameters long; gubernaculum slender consisting of two detached lateral pieces tapering distally; 10–13 papilliform precloacal supplements in two groups, the posterior three supplements smaller and closer to each other, remaining supplements larger and widely spaced; tail conico-cylindrical with half cylindrical portion. The new species is most similar to *H.sonorus* Belogurov & Fadeeva, 1980 in body shape and number of precloacal supplements, but differs from the latter species by numbers of amphid turns (2.5–3 vs 4–4.2), different shape and structure of spicules (spicules without capitulum, distal hook and central spacer vs with weak capitulum, distal hook and central spacer). The new species is also similar to *H.stagnalis* Gagarin & Long, 2017 in the number of the amphidial turns and tail, but can easily be distinguished from the latter by the different arrangement of precloacal supplements (3 posterior supplements smaller and closer vs 5–6 smaller and closer supplements

#### ﻿ Halichoanolaimus zhangi sp. nov.

Taxon classificationAnimaliaChromadoridaSelachinematidae

9ED28E12-F469-5F1C-AE60-858338D7390C

https://zoobank.org/CDC5A1AD-A09C-4716-A0DB-5D5336EE0B10

**Figure 3.**
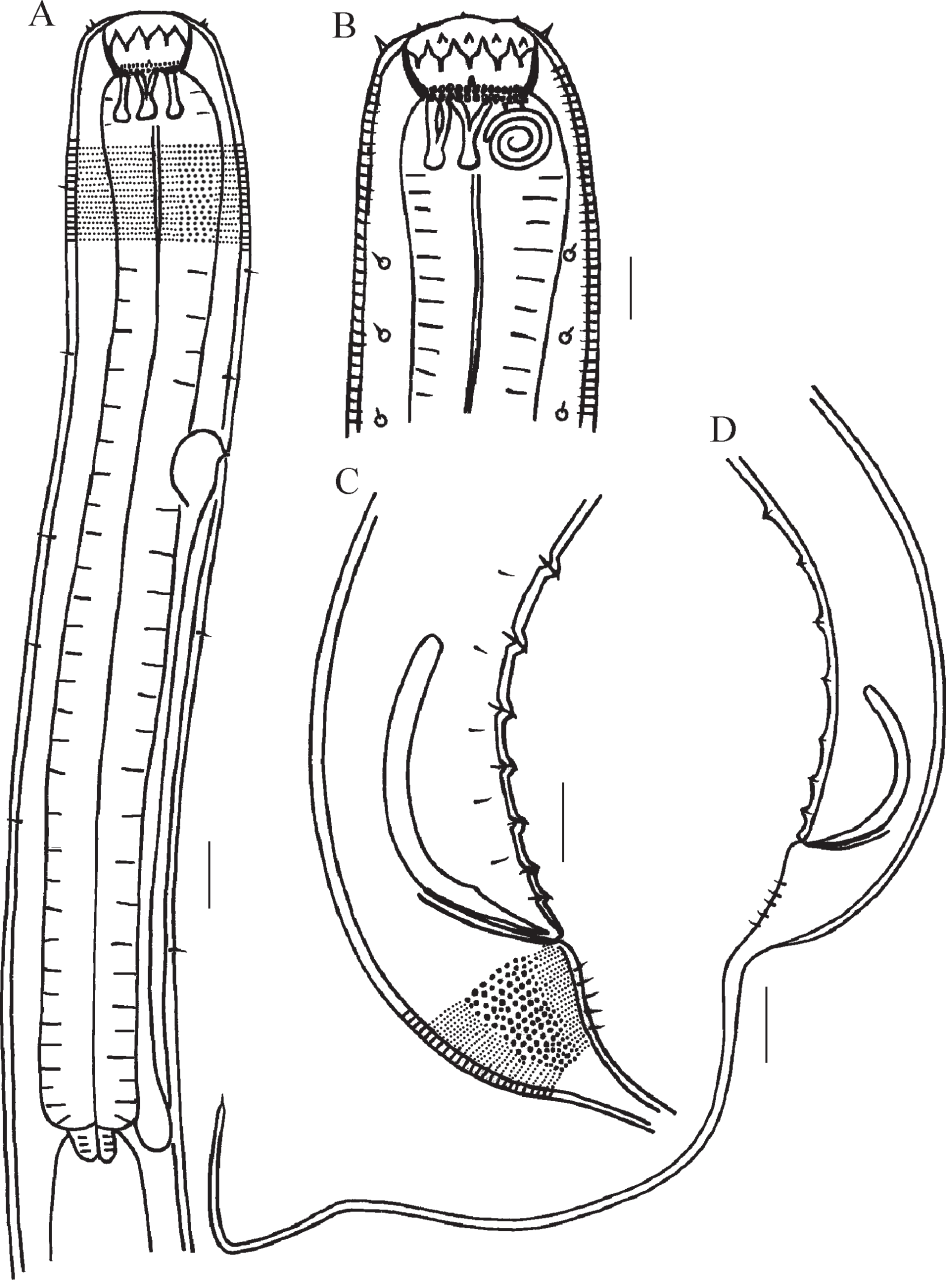
Drawings of *Halichoanolaimuszhangi* sp. nov. **A** pharyngeal region of holotype **B** anterior end of holotype **C** cloacal region of holotype **D** posterior end of male 2. Scale bars: 30 μm (**A, B, C**); 50 μm (**D**).

**Figure 4.**
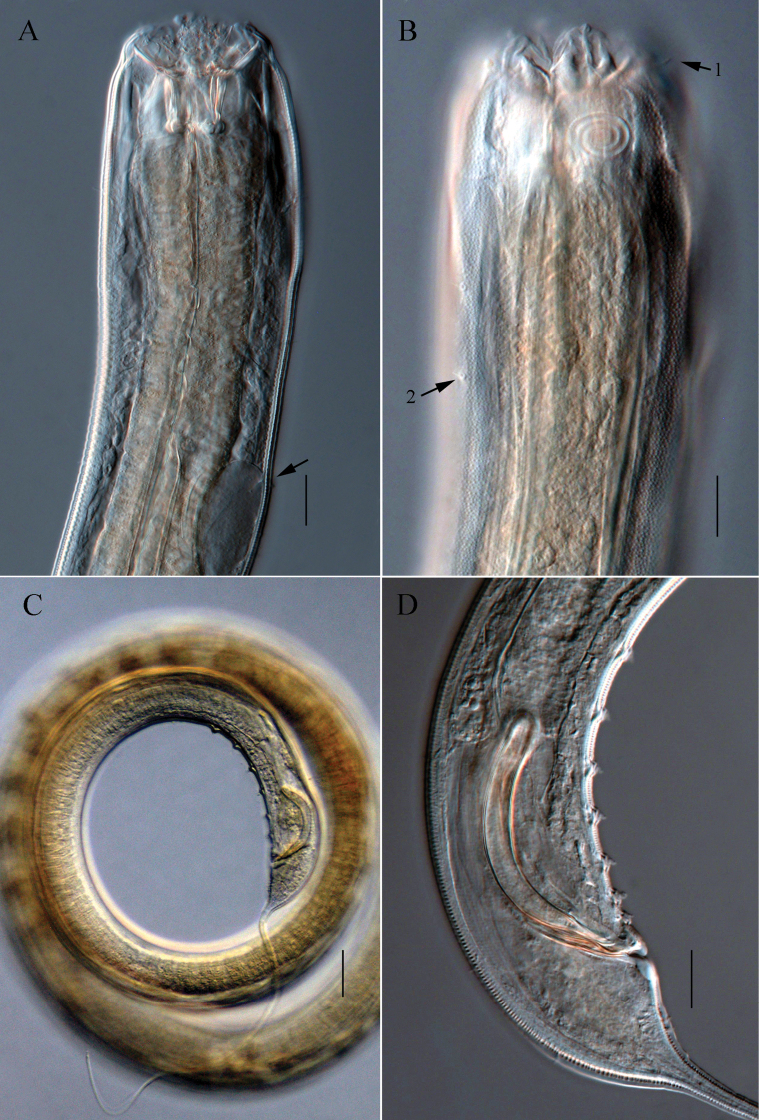
Microscopic images of *Halichoanolaimuszhangi* sp. nov. **A** anterior end of holotype, showing buccal cavity and excretory pore (arrow) **B** anterior end of holotype, showing cephalic seta (arrow 1) amphidial fovea and cuticle pores (arrow 2) **C** posterior portion of male 2, showing spicules and tail **D** cloacal region of holotype, showing spicules, gubernaculum and supplements. Scale bars: 20 μm (**A, B, D**); 50 μm (**C**).

**Figure 5.**
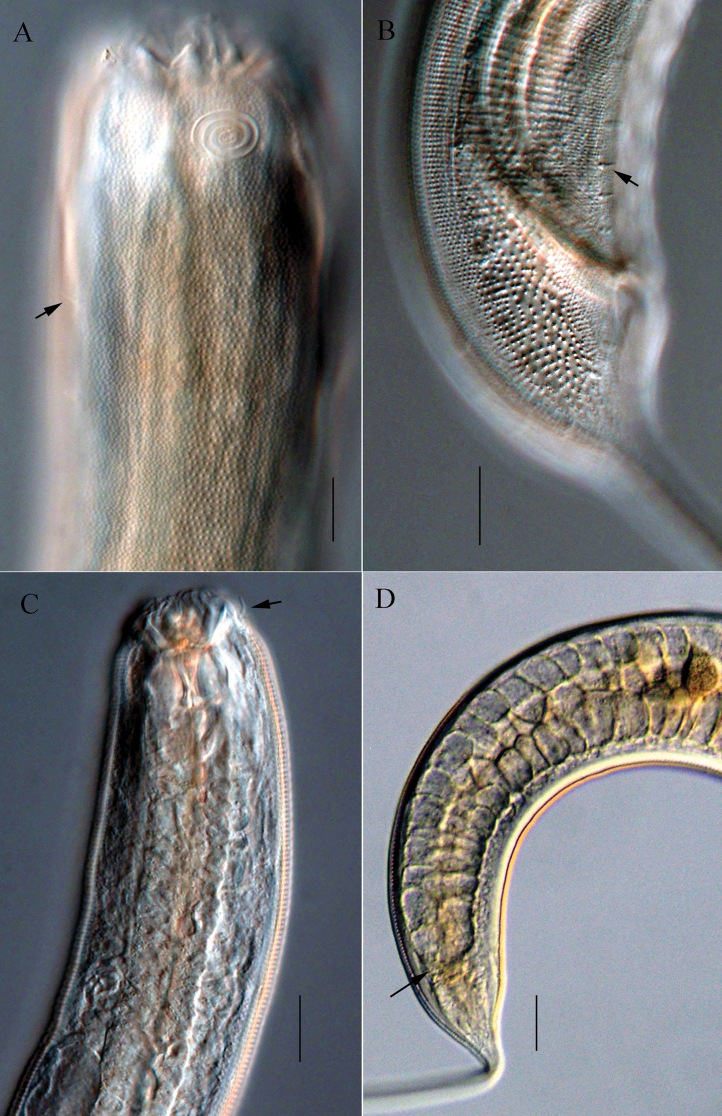
Microscopic images of *Halichoanolaimuszhangi* sp. nov. **A** anterior end of holotype, showing lateral differentiation and somatic setae (arrow) **B** cloacal region of holotype, showing lateral differentiation and conical setae situated at two subventral sides of precloacal supplements (arrow) **C** anterior end of juvenile, showing buccal cavity and cephalic sensilla (arrow) **D** posterior portion of juvenile, showing the end of intestine (arrow). Scale bars: 20 μm (**A, B, C, D**).

Figs 3 , 4 , 5 , Table 2

##### Material examined.

Two males and one juvenile were obtained. ***Holotype***: ♂1 on slide 22HSB-11-2-1; ***paratypes***: ♂2 and juvenile on slide 22HSB-11-2-2. Type specimens were deposited in the Marine Biological Museum of the Chinese Academy of Sciences, Qingdao.

##### Type locality and habitat.

Holotype and paratypes were found from intertidal muddy sediment at Rizhao coast along the Yellow Sea; 35°18'N, 119°31'E; 0–2 cm sediment depth.

##### Etymology.

The specific epithet “zhangi” is in honor of Professor Zhinan Zhang, a Chinese nematologist, in recognition of his contributions to nematode taxonomy.

##### Measurements.

All measurement data are given in Table 2.

**Table 2.** Individual measurements of *Halichoanolaimuszhangi* sp. nov. (in µm except for ratios; -, null).

Characters	Holotype	Paratypes	
	♂1	♂2	Juvenile
Total body length	3090	3075	1940
Maximum body diameter	80	79	62
Head diameter	63	60	35
Length of cephalic sensilla	5	4	5
Depth of buccal cavity	43	45	32
Width of buccal cavity	32	31	22
Width of amphid	20	18	12
Amphid from anterior end	27	28	21
Length of pharynx	483	474	332
Body diameter at pharyngeal base	78	76	60
Spicule length along arc	115	106	–
Length of gubernaculum	42	40	–
Number of precloacal supplements	7	7	–
Body diameter at cloaca	58	52	–
Tail length	630	590	314
a	38.6	38.9	31.3
b	6.4	6.5	5.8
c	4.9	5.2	6.2
c′	10.9	11.3	9.2

##### Description.

**Males.** Body cylindrical, tapering slightly towards posterior end. Cuticle with transverse rows of punctations. Lateral differentiation presents except to anterior half of pharynx with even punctations. Lateral differentiation consisting of slightly larger and more widely spaced punctations. Four longitudinal rows of pore complexes situated at sublateral sides of pharyngeal region (Fig. 4B). Each row with 10–12 pores. Cephalic region slightly rounded. Inner and outer labial sensilla papillose; four cephalic sensilla setiform, 4–5 µm long. Amphideal fovea multispiral with 3–3.25 turns, located at the level of the buccal cavity base, 27–28 µm from anterior end of body. Buccal cavity large, 43–45 µm deep, divided into anterior and posterior portions by two rows of 25 denticles. Anterior portion of buccal cavity cup-shaped, with three sets of three cuticularized rhabdions, terminating in three sets of paired denticles; posterior portion of buccal cavity narrower, cylindrical, surrounded by three Y-shaped pairs of cuticularized rhabdions with swollen bases, ca 20 µm long. Pharynx cylindrical, muscular, without anterior or posterior bulb; pharyngeal lumen cuticularized. Nerve ring difficult to distinguish. Secretory-excretory system present. Ventral gland small, situated at level of cardia; ampulla large, excretory pore situated at position of three corresponding body diameters from the anterior end. Cardia small, partially surrounded by intestine.

Reproductive system diorchic with two opposed, outstretched testes. Anterior testis to the right or ventrally to intestine, posterior testis to the left side of intestine. Spicules paired, curved, gradually narrowing from proximal to distal end with pointed tip, ca 2 cloacal body diameters long. Gubernaculum rod-like, adjoining to the dorsal side of spicules. 7 papilliform precloacal supplements, the most posterior supplement located 14 µm from the cloaca; remaining supplements gradually increasing the spacing distance forward, from 9 µm to 26 µm to each other. Each supplement consists of conical papilla and an internal duct. Two rows of short conical setae situated at two subventral sides of the precloacal supplements region of body. Tail conical with a long posterior filiform portion, accounts for 92% total tail length. A row of 5 caudal setae distributed at ventral side of tail conical part, 3–4 µm long. Caudal glands and spinneret present, 7 µm long.

Female not found. Juvenile similar to males in body shape, and with a long filiform tail, except body size smaller, intestine with blind end.

##### Differential diagnosis and discussion.

*Halichoanolaimuszhangi* sp. nov. is distinct by lateral differentiation presenting posterior to the middle of the pharynx, amphideal fovea with 3.0–3.3 turns, spicules curved, gradually narrowing from proximal to distal end with pointed tip, 7 papilliform precloacal supplements, gradually increasing the spacing distance forward, two rows of subventral stout setae situated at precloacal region, tail elongated, filiform. The new species is most similar to *H.possjetiensis* Belogurov & Fadeeva, 1980 in the filiform tail and number of precloacal supplements, but differs from the latter species by body slender (a=38.6–38.9 vs body stout, a=25.8–26.6), longer tail (c=4.9–5.2 vs c=8–8.3) and different gubernaculum (slender and straight vs. broad and hooked proximally in *H.possjetiensis*).

### ﻿Updated key to valid species of *Halichoanolaimus* (based on Zograf et al. 2015)

**Table:** 

1	Tail consists of anterior conical and posterior filiform parts	**2**
–	Tail with conical or finger-shaped (not filiform) posterior part	**25**
2	Index F (ratio of the filiform portion to the total tail length) ≤ 75%	**3**
–	Index F ≥ 80%	**16**
3	More than 5 amphid turns	**4**
–	Equal to or less than 5 amphid turns	**7**
4	5.5 amphid turns, 4 precloacal supplements	***H.macrophallus***
–	6.0–6.5 amphid turns, 3 or 5 precloacal supplements	**5**
5	5 precloacal supplements	***H.brandtae***
–	3 precloacal supplements	**6**
6	6.0 amphid turns, index c′ > 5.2	***H.anisospermus***
–	6.5 amphid turns, index c′=4.7	***H.pumilus***
7	4.5–5.0 amphid turns	**8**
–	3.0–4.2 amphid turns	**11**
8	6–8 supplements	**9**
–	2–4 supplements	**10**
9	c′=3.6, spicules 60 µm long	***H.ovalis***
–	c′=5.1–6.3, spicules 97 µm long	***H.funestus***
10	c′=3.7–5.2, spicules 43 µm long	***H.consimilis***
–	c′=2.4–3.2, spicules 56–59 µm long	***H.ossilagulus***
11	3–6 posterior-most precloacal supplements smaller and closer to each other than remaining supplements	**12**
–	All precloacal supplements almost equal in size	**14**
12	3.5–3.8 amphid turns, tail with 3/4 cylindrical portion	***H.sicaoensis***
–	2–3 amphid turns, tail with 1/2–2/3 cylindrical portion	**13**
13	Posterior 5–6 supplements smaller and closer, spicules 72–74 µm	***H.stagnalis***
–	Posterior 3 supplements smaller and closer, spicules 76–86 µm	***H.sinensis* sp. nov.**
14	Amphid with 3.2 turns, 13 supplements, spicules 73 µm long	***H.unicus***
–	Amphid with 3.5–4.2 turns, 11–12 supplements	**15**
15	Spicules 83–100 µm, 12 supplements	***H.duodecimpapillatus***
–	Spicules 63–75 µm, 11 supplements	***H.sonorus***
16	Index c = 3, male body less than 1 mm, supplements and stoma arm absent, spicules 20 µm long	***H.minutissimus***
–	Index c > 3, males body longer than 1 mm, supplements present, stoma armed, spicules longer than 40 µm	**17**
17	Index c > 14, amphid with 1.5–2.0 turns	***H.raritanensis***
–	Index c < 10, amphid with 2–7 turns	**18**
18	Dotted denticles posterior to comb in stomatorhabdion, spicules longer than 90 µm	**19**
–	Dotted denticles absent, spicules shorter than 90 µm	**21**
19	Buccal cavity armed with comb consisting of 10 denticles, 5 precloacal papillae	***H.balochiensis***
–	Buccal cavity armed with comb consisting of more than 20 denticles, 7 precloacal papillae	**20**
20	Body stout, a=26–27, c=8	***H.possjetiensis***
–	Body slender, a=39, c=5	***H.zhangi* sp. nov.**
21	Amphidial fovea 40–50% cbd, precloacal supplements barely visible	***H.minor***
–	Amphidial fovea less than 33% cbd, precloacal supplements prominent	**22**
22	Comb of each stomatorabdion armed with 7 denticles	***H.lanceolatus***
–	Comb of each stomatrhabdion armed with 9–15 denticles	**23**
23	Spicules 48–56 µm long, 6–9 precloacal supplements	***H.dolichurus***
–	Spicules longer than 70 µm	**24**
24	Index F = 96%, 4 precloacal supplements	***H.chordiurus***
–	Index F < 96%, 11–14 precloacal supplements	***H.quattuordecimpapillatus***
25	Tail with finger-shaped posterior part	***H.macrospiculatus***
–	Tail conical	**26**
26	Amphidial fovea 60% cbd, with 6 turns	***H.norvegicus***
–	Amphidial fovea more than 60% cbd, more or fewer than 6 turns	**27**
27	Index c = 13, spicules 29 µm long	***H.robustus***
–	Index c ≥ 16, spicules 50 µm or longer	**28**
28	Index c = 17, 2 supplements, spicules 50 µm long	***H.caucasicus***
–	Index c = 18–24, 5–14 supplements spicules 76–78 µm long	**29**
29	5 supplements, spicules not cephalated, with groove in the posterior third	***H.lukjanovae***
–	13–14 supplements, spicules cephalated, without groove	***H.bispirae***

## Supplementary Material

XML Treatment for Halichoanolaimus

XML Treatment for Halichoanolaimus sinensis

XML Treatment for Halichoanolaimus zhangi

## ﻿Additional information

### Author ORCIDs

Mian Huang https://orcid.org/0000-0003-3343-1520

Hongxiu Zhai https://orcid.org/0000-0003-0003-9549

### Data availability

All of the data that support the findings of this study are available in the main text.
